# The Effect of Traditional Chinese Medicine on Postviral Olfactory Dysfunction: A Systematic Review and Meta-Analysis

**DOI:** 10.1155/2023/7448034

**Published:** 2023-01-18

**Authors:** Fangfang Ma, Hewei Zhang, Bingxue Li, Duanhong Yang, Peiyu Cheng, Mingwei Yu, Dechun Dai, Xun Li, Xiaomin Wang

**Affiliations:** ^1^Beijing Hospital of Traditional Chinese Medicine, Capital Medical University, Beijing 100010, China; ^2^Department of Acupuncture-Moxibustion, Tuina and Rehabilitation, Kunshan Hospital of Traditional Chinese Medicine, Kunshan 215300, Jiangsu, China; ^3^The First Affiliated Hospital of Soochow University, Suzhou 215006, China; ^4^Ruijin Hospital, Shanghai Jiaotong University School of Medicine, Shanghai 200025, China; ^5^School of Traditional Chinese Medicine, Beijing University of Chinese Medicine, Beijing 100026, China

## Abstract

**Objective:**

The aim of this study is to evaluate the efficacy and safety of traditional Chinese medicine (TCM) for postviral olfactory dysfunction (PVOD).

**Methods:**

PubMed, EMBASE, Cochrane Central Register of Controlled Trials, China Network Knowledge Infrastructure (CNKI), Chinese Scientific Journal Database (VIP), Chinese Biomedical and Medical (CBM) Database, and Wanfang Database were electronically searched from their inception to July 25, 2022. Two authors independently performed study selection, data extraction, and quality assessment to ensure systematic quality evaluation. Randomized controlled trials (RCTs) comparing TCM with olfactory training and/or drug therapy (OTDT) were included. The outcomes were the effective rate, QOD-P, TDI score, UPSIT score, and adverse effects. Cochrane RoB was the guideline used to evaluate the methodological quality of the included trials. RevMan 5.3 software was used for statistical analysis.

**Results:**

A total of 6 RCTs involving 467 patients with PVOD were selected. Compared with OTDT, TCM plus OTDT decreased QOD-P (MD = −1.73, 95% CI (−2.40, −1.06), *P* < 0.0001) but did not increase the effective rate (T&T) (RR = 1.28, 95% CI (0.86, 1.90), *P*=0.22, *I*^2^ = 61%). Compared with no treatment, TCM seemed to increase the treatment success rate (UPSIT) (RR = 3.17, 95% CI (1.78, 5.65), *P* < 0.0001, *I*^2^ = 0%), but there was no statistically significant difference in improving the UPSIT score (MD = 3.44, 95% CI (−1.36, 8.24), *P*=0.16). Compared with drug therapy, TCM plus drug therapy appeared to increase the effective rate (ΔVAS) (RR = 2.36, 95% CI (1.41, 3.94), *I*^2^ = 0%), but there was no statistically significant difference in improving the TDI score (MD = 2.10, 95% CI (−1.99, 6.19), *P*=0.31). No significant differences in adverse reactions were reported between TCM and OTDT.

**Conclusion:**

TCM may be an effective treatment for PVOD. With a lack of high-quality RCTs, further large-scale and high-quality RCTs are still warranted.

## 1. Introduction

Olfactory dysfunction (OD) may arise from disparate mechanisms, broadly categorized as sensorineural (including head trauma, neurodegenerative disorders, and chemical injury), conductive (chronic sinusitis with nasal polyps, central compartment airway disease), or mixed. Sensorineural OD may also occur as a sequela of a viral upper respiratory tract infection (URTI), termed postviral olfactory dysfunction (PVOD) [1]. PVOD mainly consists of a decrease or loss of smell (hyposmia or hypogeusia) or absent function (anosmia or ageusia), distorted (parosmia or parageusia) or putrid sensations (cacosmia or cacogeusia), and even hallucinations (phantosmia or phantogeusia) [2, 3].

As one of the most common causes of OD, PVOD is an infection caused by several respiratory viruses, including parainfluenza virus, human coronavirus, and rhinovirus [4–6]. The varying severity of PVOD is proposed to reflect the degree of epithelial destruction, viral load, and viral serotype [6]. PVOD has a long-term impact on the quality of life of infected patients, which is associated with depressive symptoms [7], malnutrition [8], cognitive decline [9], and mortality [10].

Before the emergence of SARS-CoV-2, 18% to 42% of patients with OD had been associated with a preceding viral upper respiratory infection [11]. Since the outbreak of the COVID-19 pandemic, PVOD is becoming more apparent in this population, often in otherwise asymptomatic patients [12]. Change in the sense of smell and taste is highly prevalent in patients with COVID-19, with 40–50% of people on average reporting these symptoms globally [13, 14], and up to 98% showing olfactory dysfunction when tested objectively [15]. A meta-analysis has shown that an estimated 74%, 86%, 90%, and 96% of patients have self-reported smell recovery at 30, 60, 90, and 180 days, respectively. Meanwhile, 5% of patients with olfactory dysfunction have been lasting for more than six months after COVID-19 infection [16]. With more than 550 million people worldwide confirmed as having COVID-19 as of July 2022, of whom about 50% report smell or taste dysfunction, 5.6% of patients with persistent smell dysfunction that translates to more than 15 million patients with long-term smell dysfunctions [14].

The continuing spread of SARS-CoV-2 has caused a large burden of the disease across the world [17]. Infected patients have been facing long-term sequelae, repeated infections, repetitive positive, and prolonged or intermittent incubation periods [18]. PVOD usually follows the onset of respiratory symptoms and is associated with the inflammatory change in the respiratory mucosa and mucous discharge [11, 19, 20]. The current understanding and sequence of olfactory dysfunction following a viral upper respiratory infection (URI) begin as nasal mucosal inflammation, disrupting natural airway conduction within the nasal cavity and inhibiting the delivery of odorants to the olfactory epithelium. The persistence of olfactory dysfunction following recovery from the URI is likely explained by direct damage to the olfactory epithelium and olfactory bulb by the virus itself [6].

There are a few research studies to explore the treatment of PVOD in the COVID-19 event. Olfactory training is recommended as a first-line therapy for the treatment of PVOD, with topical corticosteroids, sodium citrate, oral vitamin A, and traditional Chinese medicine considered optional therapies for appropriately selected patients [21–23]. However, no data are available on the efficacy of treating postviral olfactory disorders.

Traditional Chinese medicine (TCM) has been used as a healing technique in China for almost 2000 years. Olfactory disorders have been associated with lung-related pathogenesis in the traditional Chinese classic “The Yellow Emperor's Internal Canon of Medicine”[21]. TCM has accumulated rich literature and case studies, among which treatment methods mainly include TCM decoction, acupuncture, acupoint injection, and the combination of various means [24]. Acupuncture is a form of traditional Chinese medicine that has minimal side effects, is cost-effective, can be easily administered, and may serve as a useful nonpharmaceutical therapy for certain conditions. Recent acupuncture studies have shown favorable results for a variety of conditions within otolaryngology, including allergic rhinitis, chronic rhinosinusitis, tinnitus, sudden sensorineural hearing loss, postviral olfactory dysfunction, dysphonia, and posttonsillectomy pain [25]. Acupoint injection is a common clinical therapy of acupuncture, and its role is widely known and recognized in the treatment of rhinitis. The acupuncture method of acupoint injection is similar to common acupuncture, but the syringe needle is replaced by the acupuncture needle. The process of acupoint injection is that the syringe needle is injected vertically without blood refluxing, which generates needle feeling; then, the syringe needle slowly injects the solution under the skin of bilateral acupoints. As a promising method for olfactory dysfunction, TCM has the advantages of internal and external treatment, definite clinical curative effects, and no obvious adverse reactions [19, 26–31].

Few studies have been collected and evaluated on the treatment of postviral olfactory dysfunction. Therefore, this meta-analysis is used to analyze the results of relevant clinical trials and evaluate the effect of TCM on PVOD, which would provide more reliable evidence-based medical evidence for clinical practice.

## 2. Methods

### 2.1. Protocol and Registration

The protocol for this systematic review was registered in the Prospective Register of Systematic Reviews (PROSPERO): CRD42021238977.

### 2.2. Literature Search

The following databases were searched from their inception until July 25, 2022: PubMed, EMBASE, Cochrane Library, Web of Science, Science Direct, China Network Knowledge Infrastructure (CNKI), Chinese Scientific Journal Database (VIP), Chinese Biomedical and Medical (CBM) Database, and Wanfang Database. The complete manuscripts of all relevant studies published in English and Chinese were retrieved. The major search terms were Traditional Chinese medicine OR Chinese medicine OR Chinese herb OR decoction OR acupuncture OR moxibustion OR massage OR cupping OR acupoint injection) AND (viral illness OR post-viral OR virus OR viral) AND (Olfactory disorders OR Olfactory dysfunction OR Smell disorder OR Cacosmia OR Dysosmia OR Paraosmia OR Anosmia. The search strategy was modified to suit each database.

### 2.3. Inclusion and Exclusion Criteria

#### 2.3.1. Types of Studies

To collect high-quality evidence, only RCTs were included in this systematic review. Only full articles were included.

#### 2.3.2. Types of Participants

This study considered patients of any age with olfactory dysfunction attributed to viral illness without restrictions on race or gender. The diagnosis of olfactory disorder includes the collection of the patient's medical history, the examination of nasal endoscopy, and the examination of olfactory function.

Studies reporting PVOD with a history of an upper respiratory tract infection without a gap period were included, while olfactory dysfunction must be accompanied by virus infection.

Studies on all other conditions for olfactory loss attributed to previous surgery of the nose and the paranasal sinuses, exposure to toxic substances, congenital diseases, occupying lesions, acute or chronic inflammatory nasal disease, and psychiatric factors were excluded.

#### 2.3.3. Types of Intervention

*(1) Treatment Interventions*. We focused on treatment of traditional Chinese medicine combined with or without conventional treatment. We included the studies that evaluated any type of traditional Chinese medicine, such as traditional Chinese medicine decoction, acupuncture, moxibustion, massage, cupping, and acupoint injection.

*(2) Control Interventions*. We included studies recruiting patients receiving conventional treatment (western medicine and/or olfactory training, OTDT), no treatment, or placebo control to prevent postviral olfactory dysfunction as control.

#### 2.3.4. Types of Outcomes

The primary outcome was the effective rate of postviral olfactory dysfunction in the treatment:

1. Effective rate (T&T): The T&T olfactometer test was conducted to measure the sense of smell subjectively with five flavors such as fecal odor, fruit, corrupt, coke, and fragrance. Efficiency = heal + obviously effective + effective.
2. Treatment success rate (UPSIT): Olfactory function was evaluated using the University of Pennsylvania Smell Identification Test (UPSIT) before and after treatment. The sum of the test results was used as a measure of olfactory function, which allows the grouping of participants as patients (>15 scores), hyposmic (15–35 scores), and normosmic (>35 scores). Treatment success was defined as a score increased by at least four points.
3. Effective rate (ΔVAS): Olfactory function was evaluated using the Visual Analogue Scale (VAS). ΔVAS score = the VAS score before the treatment–the VAS score after the treatment. Heal: the VAS score reached 0∼1 score; obviously effective: the ΔVAS score ≥4 scores; improvement: 2 scores ≤ the ΔVAS score ≤ 3 scores; invalid: the ΔVAS score ≤1 score. Efficiency = heal + obviously effective + effective.

The secondary outcomes included the questionnaires of olfactory disorders (QOD-P), Sniffin' Sticks test (TDI score), and the UPSIT score. All side effects and adverse events reported were included as safety outcomes.

1. QOD-P: the questionnaires of olfactory disorders (parosmia statements) classified olfactory function objectively into four degrees: *P*1, *P*2, *P*3, and *P*4 (agree, partly agree, partly disagree, and disagree) according to the score (3, 2, 1, and 0).
2. TDI score: The TDI score evaluated olfactory function by Sniffin's Sticks Test before and after treatment. Odorants were presented in felt-tipped pens; for odor presentation, the cap was removed by the investigator, and the pen's tip was placed in front of the subject's nostrils for approximately 15 seconds. This test battery assessed olfactory function bilaterally and involved subtests for odor threshold (*T*), discrimination (*D*), and identification (*I*). The TDI score was used as a measure of olfactory function, which allowed grouping of patients into anosmic (TDI score <15), hyposmic (15 < TDI score < 30), and normosmic (30 < TDI score).
3. UPSIT score: Olfactory function was evaluated using the University of Pennsylvania Smell Identification Test (UPSIT) before and after treatment. The sum score of the test results was used as a measure of olfactory function.

### 2.4. Data Collection and Analysis

#### 2.4.1. Selection of Studies

**Figure 1:**
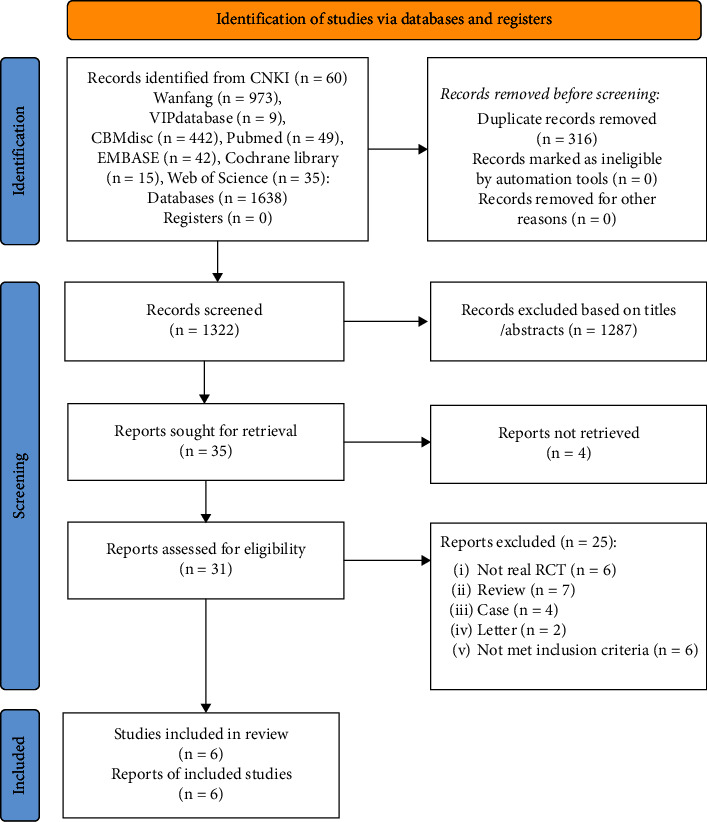
Flow diagram of the studies included in the review.

All the authors were trained regarding the purpose and process of the review. Two authors independently performed the study selection by screening the titles and abstracts of all retrieved studies and then by reading through the full text independently to decide eligibility. If any disagreement existed throughout the process, the third author made the final decision. The selection process of selecting the eligible study is shown in a Preferred Reporting Items for Systematic Review and Meta-Analysis (PRISMA) flow diagram (Figure 1).

#### 2.4.2. Data Extraction and Management

Two independent reviewers conducted data extraction based on a predesigned data extraction form. The third author settled any disagreement during the process. According to the recommendation of the Cochrane Handbook, all extracted data and information management were recorded in an Excel extraction form.

The following data were extracted:

1. Basic information of the study: title, first author's name, year of publication, country, and journal.
2. Participants' characteristics: age, sex, number of participants, disease, inclusion criteria, exclusion criteria, and baseline situation.
3. Interventions: details of TCM (such as treatment methods, sessions, and frequency), treatment duration, study design, randomization, allocation concealment, and blinding methods.
4. Comparators: western medicine or/and olfactory training.
5. Outcomes: measures and primary and secondary outcomes.

#### 2.4.3. Assessment of Risk of Bias

The Cochrane Collaboration's ‘risk of bias” assessment tool was used to assess the potential source of bias in the included studies. Two authors first evaluated the risk of bias in eligible studies separately and then cross-checked their findings. This quality assessment was based on random sequence generation, allocation concealment, blinding of participants and personnel, blinding of outcome assessment, incomplete outcome data, selective reporting, and other biases. We graded the risk of bias in the included trials and classified it into 3 levels: “high risk of bias,” “low risk of bias,” and “unclear risk of bias.” The third author resolved any disagreement and made a final decision.

#### 2.4.4. Measures of Treatment Effects

The available data on treatment outcomes were extracted and meta-analyzed. The weighted mean difference (MD) or standardized mean difference (SMD) with 95% CIs was used for continuous data. The risk ratio (RR) with its 95% CI was used for dichotomous data.

#### 2.4.5. Dealing with Missing Data

If the presented data of the study were inconsistent or missing, we tried to contact the corresponding author or the relevant author for the required data via email. Otherwise, the study was excluded.

#### 2.4.6. Data Synthesis

RevMan software (V.5.3) was used to complete the data analysis and synthesis by the Cochrane Collaboration. If little heterogeneity existed among the trials, a fixed-effect model was established, while a random-effect model was carried out for significant heterogeneity. Dichotomous data were analyzed by the risk ratio (RR) with 95% CIs, and continuous data were analyzed by the mean difference (MD) or standard MD (SMD) with 95% CIs.

### 2.5. Assessment of Heterogeneity

Statistical heterogeneity was assessed in the forest plot if data from more than 10 studies were pooled, and it was detected by the standard *X*^2^ test and the *I*^2^ test. The interpretations of the *I*^2^ test are as follows: (1) 0% to 40%: might not be important; (2) 30% to 60% may represent moderate heterogeneity; (3) 50% to 90% may represent substantial heterogeneity; and (4) 75% to 100%: considerable heterogeneity.

### 2.6. Subgroup Analysis

If obvious heterogeneity existed in a single meta-analysis, subgroup analysis was conducted to analyze the heterogeneity of available data according to the change in characteristics of trial participants, type of traditional Chinese medicine, and type of conventional treatment.

### 2.7. Sensitivity Analysis

Sensitivity analysis was needed to evaluate the robustness and reliability of the result when sufficient data existed. We conducted the sensitivity analysis in two ways: (1) exclude any of the studies; (2) change the effect model to verify the synthesized result. When a low-quality study was identified and excluded, the meta-analysis pertained to low heterogeneity. A certain result could determine whether a low-quality study should be included or not. The final result depended on the sample size, missing data, risk of bias, and quality of methods of each study.

### 2.8. Certainty Assessment

The Grading of Recommendation, Assessment, Development, and Evaluation (GRADE) system was used to assess the quality of evidence. Two investigators performed the assessment independently and gave a summary of the finding of the table together. The third person would be necessary when there was any disagreement between the two investigators.

### 2.9. Ethics and Dissemination

Since we performed a secondary analysis of the published article, ethical approval was not required. The results will be published in a peer-reviewed journal and presented at a relevant conference.

## 3. Results

### 3.1. Literature Search Results

A total of 1638 studies were retrieved in the initial search. After removing 316 duplicates, 1322 studies were identified for further analysis. Through screening the title and abstract, 1287 studies were excluded because they were literature reviews, case reports, letters, duplicates, or irrelevant studies. Of the remaining 35 studies, 6 studies met our inclusion criteria by reading the full text, as shown in Figure 1.

### 3.2. Characteristics of the Included Studies

The basic characteristics of the included studies are listed in Table 1 [32–37]. By integrating 6 included studies, a total of 467 cases were reported, with 233 cases in the treatment group and 234 cases in the control group. The publication period was from 2010 to 2020. Two studies were published in English, and the other 4 studies were published in Chinese. All of the included patients were concentrated in China with a large difference in ages (18–80 years); meanwhile, the overall patients in the 2 groups were mainly balanced in terms of age, mean outcome measures, and the distribution of disease baseline.

### 3.3. Risk of Bias and Assessment of the Quality

**Figure 2:**
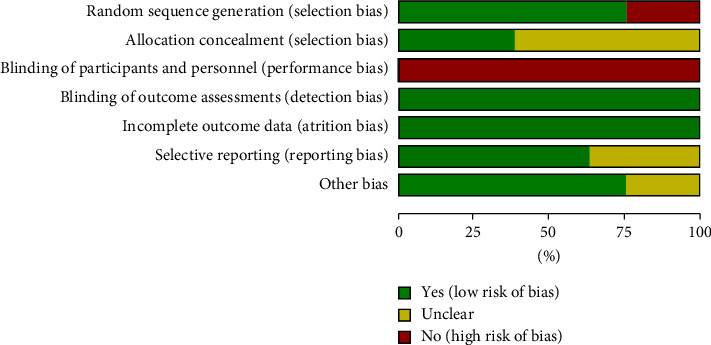
Risk of bias graph.

**Figure 3:**
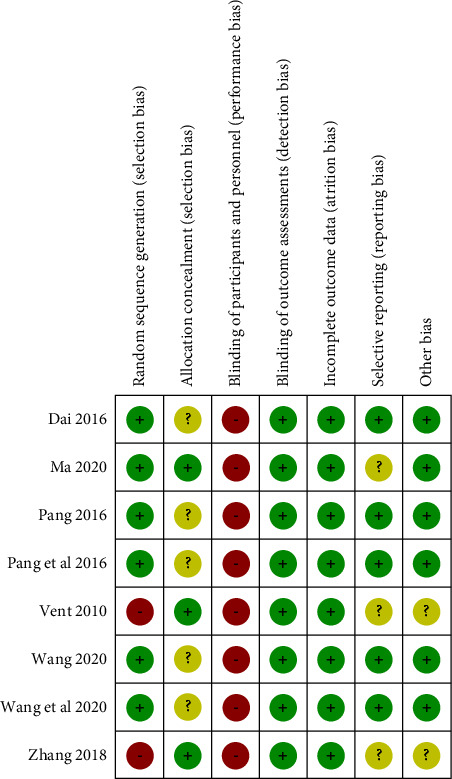
Risk of bias summary.

According to the Cochrane Handbook for Systematic Reviews of Interventions, we assessed the risk of bias in the included literature. The details of the risk of bias (ROB) assessment are provided in Figures 2 and 3. Six RCTs described the appropriate random sequence generation method in detail. The other 2 RCTs were historical cohort studies in which random sequence generation was assessed to be at high ROB. Three studies reported allocation with low ROB, and 5 studies did not report the allocation concealment with unclear ROB. Due to the nature of traditional Chinese medicines like acupuncture and acupoint injection not blinding participants and personnel, all studies were assessed to be at high ROB. All of the included studies reported the complete outcome data, and we considered them to be at low ROB. Three studies reported the details of adverse effects or published protocols with low ROB, while the other 5 studies did not report them with unclear ROB. Six studies reported the patients' baseline characteristics with low ROB based on other sources of bias. The other 2 studies were judged to be at unclear ROB due to lack of reporting details. The overall quality of the trials was assessed as a moderate risk of bias.

### 3.4. Effectiveness Assessment

#### 3.4.1. Primary Outcome Measure: The Effective Rate of PVOD

**Figure 4:**
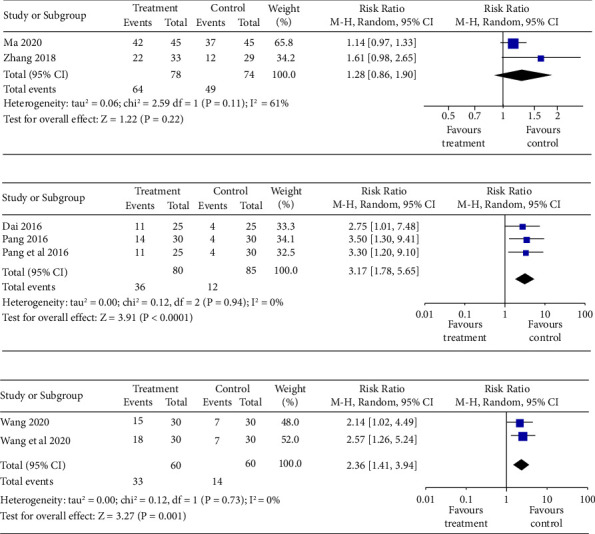
(a) Meta-analysis forest map of the effective rate (T&T). (b) Meta-analysis forest map of treatment success rate (UPSIT). (c) Meta-analysis forest map of the effective rate (ΔVAS).

1. *Effective Rate (T&T).* Two studies [33, 35] showed no significant difference in the effective rate (T&T) of PVOD between TCM + OTDT and olfactory training and/or drug therapy (OTDT) (*n* = 152, RR = 1.28, 95% CI (0.86, 1.90), *P*=0.22) with high heterogeneity (*P*=0.11, *I*^2^ = 61%), as shown in Figure 4(a).
2. *Treatment Success Rate (UPSIT).* Three studies [32, 37]showed a significant difference in the treatment success rate (UPSIT) between TCM and no treatment (*n* = 165, RR = 3.17, 95% CI (1.78, 5.65), *P* < 0.0001) with low heterogeneity (*P*=0.94, *I*^*2*^ = 0%), as shown in Figure 4(b).
3. *Effective Rate (ΔVAS).* Two studies [34] showed a significant difference in the effective rate (ΔVAS) between TCM with drug therapy and drug therapy (*n* = 120, RR = 2.36, 95% CI (1.41, 3.94), *P*=0.001) with low heterogeneity (*P*=0.73, *I*^*2*^ = 0%), as shown in Figure 4(c).

#### 3.4.2. Secondary Outcomes

**Figure 5:**
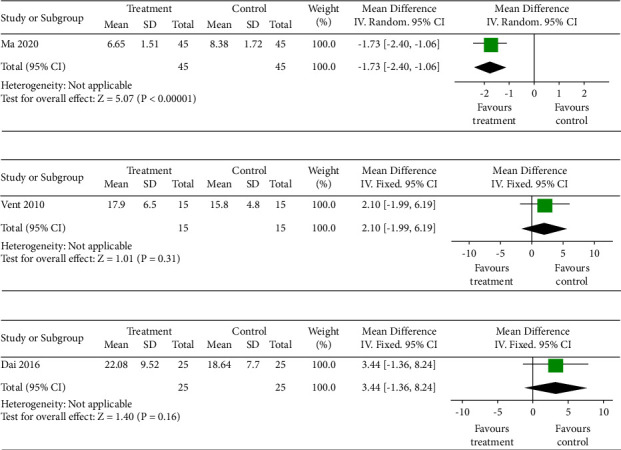
(a) Meta-analysis forest map of QOD-P. (b) Meta-analysis forest map of the TDI score. (c) Meta-analysis forest map of the UPSIT score.

1. *Questionnaires of Olfactory Disorders (QOD-P).* One study [33] showed a significant difference in QOD-P between TCM + OTDT and olfactory training and/or drug therapy (OTDT) (*n* = 90, MD = −1.73, 95% CI (−2.40, −1.06), *P* < 0.0001), as shown in Figure 5(a).
2. *Sniffin's Sticks Test (TDI score).* One study [36] showed no significant difference in the TDI score between TCM with drug therapy and drug therapy (*n* = 30, MD = 2.10, 95% CI (−1.99, 6.19), *P*=0.31), as shown in Figure 5(b).
3. *UPSIT Score.* One study [37] showed no significant difference in the TDI score between TCM and no treatment (*n* = 50, MD = 3.44, 95%CI (−1.36, 8.24), *P*=0.16), as shown in Figure 5(c).
4. *Adverse Event.* Three studies [34, 36, 37] investigated adverse events, whereas the other 4 studies did not report any adverse event. Two studies [36, 37] showed that no harm or adverse events were experienced during and after the treatment between the two groups. One study [34] reported a total of 9 mild adverse events in 90 cases including dizziness (*n* = 2) and pain (*n* = 2) in the treatment group and stomach discomfort (*n* = 5) in the control group treated with sleeping pills, and adverse events in the two groups were improved after symptomatic treatment. Because of the lack of adequate studies, there exist no significant differences reported between TCM treatment and olfactory training and/or drug therapy on PVOD.

### 3.5. Sensitivity Analysis

Sensitivity analysis was not available as there were inadequate data in the meta-analysis.

### 3.6. Quality of Evidence

**Figure 6:**
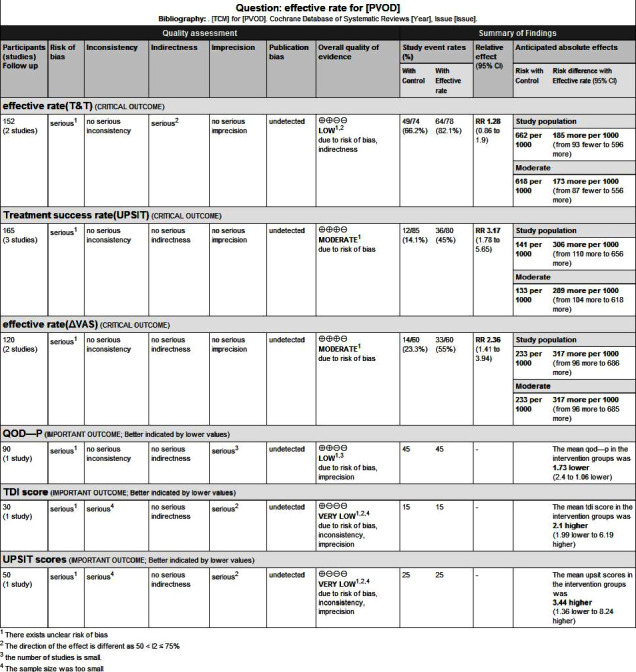
The summary findings by the Grading of Recommendations, Assessment, Development, and Evaluation (GRADE) methods.

The certainty of evidence for the outcomes of the meta-analysis was assessed by the Grading of Recommendation, Assessment, Development, and Evaluation (GRADE) method, and it showed that the quality of evidence for the results of the meta-analysis ranged from “very low” to “low.” Since the nature of traditional Chinese medicine as acupuncture and acupoint injection could not meet blinding of participants and personnel, heterogeneity existed in the intervention. The sample size was small, so some studies showed no significant difference between TCM and no treatment. The main reasons for the downgrade were the small sample size and the unclear risk of bias in the selected studies, as shown in Figure 6.

## 4. Discussion

The pathophysiology of PVOD is not clear, and its methods of treatment consist of corticosteroids, supplementation with vitamin B complex, olfactory training, and others [36]. Studies showed that the human olfactory nervous system could be reshaped. Olfactory sensory neurons in the olfactory mucosa had the characteristics of sustainable regeneration lifetime in the nervous system of all vertebrates. Olfactory training could repeatedly stimulate the olfactory epithelium and the olfactory pathway through various olfactory elements so that the damaged olfactory function could be improved or restored [38–41]. The systemic glucocorticoid and the *Ginkgo biloba* extract were recommended, but the curative effect was uncertain [22]. One study showed that the total effective rate of PVOD patients treated with nasal pneumatic spray aerosol-inhaled budesonide suspension was 90%, but most of the patients (65%) did not return to normal [42]. There were few effective ways of western medicine to treat PVOD.

There are a large number of records about the treatment of “no smell of the nose” documented in traditional Chinese medical classics. A wealth of treatment information has been accumulated, not only on internal clothing but also on various external treatments such as the stuffing nose, the blowing nose, nasal irrigation, acupuncture, and massage. Acupuncture and moxibustion theory have been proved effective in postviral olfactory dysfunction [43], and they could improve the olfactory sensitivity of healthy people [44]. The decoction of TCM, like “Danggui Shaoyao powder” and “Ginseng Yangrong decoction,” has been proved more effective than nasal glucocorticoids. 43% of patients treated with Danggui Shaoyao powder and 36% of patients treated with Ginseng Yangrong decoction, respectively, improved olfactory disorders after viral infection [45].

The aim of this study was to evaluate the effectiveness and safety of TCM in patients with PVOD. Based on the included studies, we pooled the data from 6 studies involving 467 patients for further analysis. Our pooled analysis indicated that, compared with conventional treatment (western medicine or/and olfactory training), TCM seemed to improve postviral olfactory dysfunction measured by QOD-P and increase the effective rate of PVOD with mild adverse events. As treatment success was defined as a score increase by at least four points, the UPSIT score [37] (RR = 3.44, 95% CI (−1.36, 8.24), *P*=0.16) or the TDI score [43] (RR = 2.10, 95% CI (−1.99, 6.19), *P*=0.31) in this study could not meet the standard of treatment success. Due to the small number of included studies, further research needs to enlarge the sample size.

The common acupoints of the acupuncture for treatment of PVOD in the included studies were LI20, EX-HN8, EX-HN3, LG20, GV23, BL7, LI4, Liv3, SP10, ST36, LG16, LU7, LU9, KD3, BL2, and BiQiu (EX-HN). As extra nerve points, BiQiu (EX-HN) was located at the anterior end of the middle turbinate in the lateral wall of the nasal cavity that preserves the nerve, and sphenopalatine nerve branches were sensitive parts of the nasal cavity and the target organs of allergic rhinitis. The needle frequency of the treatment of PVOD was 1 time a day for 3 weeks or 3 times a week for 3 months. The common acupoints of the acupoint injection for treatment of PVOD in this study were LI20, RN22, EX-HN8, and BiQiu (EX-HN). Needle frequency was 3 times a week. 10 times was 1 course with a treatment interval of 3 to 5 days. The total treatment duration was 3 months.

In our review, there were two common methods of TCM, acupuncture and acupoint injection treating PVOD, which had significant improvement in smell and taste function. It is believed that TCM treatment could reestablish an equilibrium for a diseased body [36]. According to Chinese acupuncture guidelines, therapy should be individualized to restore physical balance and to bring yin and yang into equilibrium.

The injection solution of acupoint injection in this study was 0.1∼0.2 ml lidocaine mixed with 1 ml vitamin B12. The injection was 0.5 ml per side. Needle frequency was 3 times a week. 10 times was 1 course with a treatment interval of 3∼5 days. The total treatment duration was 3 months. Acupoint injection therapy is a method of injecting an appropriate amount of liquid medicine into specific acupoints, combining the function of acupoints and the mechanical effect of acupuncture with the pharmacological effect of drugs to prevent and treat diseases. It has the characteristics of simple operation, safety, small dosage, rapid action, and good acceptance by patients [46].

The quality of evidence ranged from low to high based on the Cochrane Bias Evaluation Tools and the Jadad scale. Among the 6 studies included, 1 study was of high quality, 3 studies were of moderate quality, and 2 studies were of low quality. The quality of the included studies was generally moderate. The inappropriate random method, allocation concealment, and lack of blinding in most studies exaggerated the result of the outcome measures.

No statistical heterogeneity was found in the effective rate of the effectiveness of TCM treating PVOD, which may be because all the included studies explored the acupoint-stimulating therapy without traditional Chinese herbs. Missing data on Chinese herbal medicine, despite its widespread clinical use, reveal an evidence gap.

Furthermore, these studies had problems such as low quality, the flaw in test design, small sample sizes, or the variety of the intervention and control groups. To a certain extent, these indicated that the results of this meta-analysis were affected by the risk of bias.

There were several limitations in this systematic review and meta-analysis. The methodological quality of most included studies was relatively low, which resulted in the potential risk of bias. To some extent, it also weakened the credibility and reliability of the evidence of TCM for the treatment of PVOD in this systematic review and meta-analysis. For example, due to few control groups being placebo control or false TCM, it was difficult to eliminate the placebo effect. Since the nature of traditional Chinese medicine like acupuncture and acupoint injection could not meet the blinding of participants and personnel, it might lead to potential implementation bias and selection bias.

Traditional Chinese medicine has little or no side effects, and it is cost-effective and easily administered. Regardless of the potential bias and limitations of this review, all of the included studies showed that TCM intervention seemed to have significant effects in improving PVOD. As an effective adjunct treatment, TCM intervention has turned out to be a superior complementary and alternative theory, which has potential efficacy and caused few adverse effects.

## 5. Conclusion

In this systematic review and meta-analysis of RCTs, the effectiveness of traditional Chinese medicine in the treatment of postviral olfactory dysfunction is positive. TCM has the potential to become a more effective therapy than conventional treatment (western medicine or/and olfactory training). Because of the lack of adequate studies, there exist no significant differences in the adverse reactions reported between TCM treatment and conventional treatment of POVD.

In order to improve the credibility of the research and better guide clinical practice, well-designed, rigorous, large sample, and multicenter prospective RCTs are needed. The long-term effectiveness of TCM intervention in PVOD must also be evaluated.

## Figures and Tables

**Table 1:** Basic characteristics of the included studies.

Study	Sample size (T/C)	Disease course (1∼3 m/4∼6 m/>6m)	Olfactory function (N/P)		Age year (mean ± SD)	Gender male/female	Control	Intervention	Main treated acupoints and treatment frequency	Course (weeks)	Adverse events (T/C)
Pang et al. 2016 [32]	30/30	*T*: 30 (14/5/11); *C*: 30 (19/5/6)		*T*: 13/17 *C*: 16/14	*T*: 50.2 ± 14.3 *C*: 47.1 ± 12.1	*T*: 8/22 *C*: 6/24	No treatment	Acupoint injection	Acupoint injection: Yingxiang (LI20). 3 times a week for 3 months. 10 times is 1 course with a treatment interval of 3∼5 days	3 m	Treatment success rate (UPSIT)
Pang et al. 2016 [32]	25/30	*T*: 25 (10/5/10); *C*: 30 (19/5/6)		*T*: 10/15 *C*: 16/14	*T*: 54.2 ± 12.7 *C*: 47.1 ± 12.1	*T*: 11/14 *C*: 6/24	No treatment	Acupoint injection	Acupuncture:Yingxiang (LI20), Shangyingxiang (EX-HN8), BiQiu. 3 times a week for 3 months	3 m	Treatment success rate (UPSIT)
Ma and Feng 2020 [33]	45/45	*T*: 7–18 (11.7 ± 2.4) m; *C*: 5–17 (11.5 ± 2.1) m		*T*: 19/26 *C*: 17/28	*T*: 41.1 ± 3.6 *C*: 40.8 ± 3.2	*T*: 24/21 *C*: 25/20	Olfactory training + drug therapy	Acupuncture + Acupoint injection + *C*	Acupuncture: Yingxiang (LI20), Shangyingxiang (EX-HN8), Yintang (EX-HN3). 3 times a week for 3 months. Acupoint injection: Yingxiang (LI20). 3 times a week for 3 months. 10 times is 1 course with a treatment interval of 3∼5 days	3 m	Effective rate (T&T); QOD-P
Wang et al. 2020 [34]	30/30	*T*: 30 (9/10/11); *C*: 30 (11/9/10)		*T*: 12/18 *C*: 14/16	*T*: 49.7 ± 2.2 *C*: 49.3 ± 2.3	*T*: 14/16 *C*: 10/20	Drug therapy	Acupoint injection + *C*	Acupoint injection: Tiantu (RN22). 2 times a week for 3 months.8 times is 1 course	3 m	Effective rate (ΔVAS)
Wang 2020 [34]	30/30	*T*: 30 (10/7/13); *C*: 30 (11/9/10)		*T*: 14/16 *C*: 14/16	*T*: 49.8 ± 2.5 *C*: 49.3 ± 2.3	*T*: 9/21 *C*: 10/20	Drug therapy	Acupoint injection + *C*	Acupoint injection: Yingxiang (LI20). 2 times a week for 3 months. 8 times is 1 course	3 m	Effective rate (ΔVAS)
Zhang 2018 [35]	33/29	*T*: 7–22 (12.9 ± 5.6) m; *C*: 6.5–21 (13.8 ± 4.5) m		*T*: 17/16 *C*: 15/14	*T*: 40.5 ± 8.3 *C*: 41.3 ± 8.9	*T*: 12/21 *C*: 10/19	Olfactory training + drug therapy	Acupuncture + *C*	Acupuncture: Yingxiang (LI20), Shangyingxiang (EX-HN8), Cuanzhu (BL2). 3 times a week for 3 months	3 m	Effective rate (T&T)
Wilkinson 2010 [36]	15/15	4.3 y		None	*T*: 63.1 ± 6.8 *C*: 61.43 ± 8.72	*T*: 8/7 *C*: 8/7	Vitamin B complex (B1, B6, B12) oral 12 weeks	Acupuncture	Acupuncture: Fengfu (LG16), Baihui (LG20), Yingxiang (LI20), Lieque (LU7), Taiyuan (LU9), ZusanLi (ST36), TaiXi (KD3). 2 times a week for 10 weeks	10 w	Sniffin's Sticks TDI score
Dai et al. 2016 [37]	25/25	*T*: 25 (10/5/10); *C*: 25 (15/5/6)		*T*: 10/15 *C*: 11/14	*T*: 54.2 ± 12.7 *C*: 49.1 ± 12.8	*T*: 11/14 *C*: 5/20	No treatment	Acupuncture	Acupuncture: Yingxiang (LI20), Shangyingxiang (EX-HN8), BiQiu. 3 times a week for 3 months. 10 times is 1 course with a treatment interval of 3∼5 days	3 m	Treatment success rate (UPSIT); UPSIT score

*T*, the treatment group; *C*, the control group; M, months; Y, years; W, week; *N*, anosmic; *P*, hyposmic. ① The intervention acupoint injection: The injection solution is 0.1∼0.2 ml lidocaine mixed with 1 ml vitamin B12. When needle feeling is generated as the needle vertically injected without blood refluxing, the needle slowly injects the solution under the skin of bilateral acupoints. The injection is 0.5 ml per side. Needle frequency is 3 times a week. 10 times is 1 course with a treatment interval of 3∼5 days. The total treatment duration is 3 months. ② Olfactory training: Patients are advised to smell five different smells of wind oil essence, vinegar, hemp oil, alcohol, and rose fragrance for 10 seconds one time and then take 10 minute rest and smell another smell. This training has lasted 2 times a day. ③ Drug therapy: cobalt tablets (once 0.5 mg, 3 times a day) + prednisone tablets (once 30–10 mg, 1 times a day) + ginkgo leaf extract tract (once 40 mg, 3 times a day).

## Abbreviations

OD: Olfactory dysfunction
URI: Upper respiratory infection
PVOD: Postviral olfactory dysfunction
TCM: Traditional Chinese medicine
CNKI: China Network Knowledge Infrastructure
VIP: Chinese Scientific Journal Database
CBM: Chinese Biomedical and Medical Database
RCTs: Randomized controlled trials
VAS: Visual Analogue Scale
QOD: Questionnaires of olfactory disorders
OTDT: Olfactory training and drug therapy
UPSIT: University of Pennsylvania Smell Identification Test
TDI score: Sniffin's Sticks Test assessed olfactory function bilaterally and involved subtests for odor threshold (*T*), discrimination (*D*), and identification (*I*).

## Supplementary Materials

Supplementary Materials 1. *PRISMA Checklist.* The Preferred Reporting Items for Systematic Reviews and Meta-Analysis (PRISMA) is a 27-item checklist used to improve transparency in systematic reviews. These items cover all aspects of the manuscript, including titles, abstracts, introduction, methods, results, discussion, and funding.
